# Locally applied Simvastatin improves fracture healing in mice

**DOI:** 10.1186/1471-2474-8-98

**Published:** 2007-09-27

**Authors:** Björn Skoglund, Per Aspenberg

**Affiliations:** 1 Division of Orthopedics and Sports Medicine, Department of Neuroscience and Locomotion, Faculty of Health Sciences, Linköping University, SE 581 85 Linkoping, Sweden

## Abstract

**Background:**

HMG-CoA reductase inhibitors, statins, are widely prescribed to lower cholesterol. High doses of orally administered simvastatin has previously been shown to improve fracture healing in a mouse femur fracture model. In this study, simvastatin was administered either subcutaneously or directly to the fracture area, with the goal of stimulating fracture repair at acceptable doses.

**Methods:**

Femur fractures were produced in 70 mature male Balb-C mice and stabilized with marrow-nailing. Three experiments were performed. Firstly, 20 mice received subcutaneous injections of either simvastatin (20 mg) or vehicle. Secondly, 30 mice were divided into three groups of 10 mice receiving continuous subcutaneous delivery of the vehicle substance, the vehicle with 5 mg or with 10 mg of simvastatin per kg bodyweight per day. Finally, in 20 mice, a silicone tube was led from an osmotic mini-pump to the fracture area. In this way, 10 mice received an approximate local dose of simvastatin of 0.1 mg per kg per day for the duration of the experiment and 10 mice received the vehicle compound. All treatments lasted until the end of the experiment. Bilateral femurs were harvested 14 days post-operative. Biomechanical tests were performed by way of three-point bending. Data was analysed with ANOVA, Scheffé's post-hoc test and Student's unpaired t-test.

**Results:**

With daily simvastatin injections, no effects could be demonstrated for any of the parameters examined. Continuous systemic delivery resulted in a 160% larger force at failure. Continuous local delivery of simvastatin resulted in a 170% larger force at failure as well as a twofold larger energy uptake.

**Conclusion:**

This study found a dramatic positive effect on biomechanical parameters of fracture healing by simvastatin treatment directly applied to the fracture area.

## Background

In 1999, Mundy *et al *described a set of experiments, which indicated that a group of common cholesterol lowering drugs, the statins, have anabolic effects on bone[1]. Other experiments supporting this finding have followed [2-11]. However, other studies have not shown any such effect, most notably the study reported by Maritz et al, which in essence repeated the study by Mundy et al and found diametrically different results[12]. Also the experiments reported by von Stechow et al found no positive effect on undisturbed bone by simvastatin in mice[13]. Thus, there still remains some controversy concerning the effect of statins on bone formation. In 2002, the authors reported on a dramatic improvement of fracture repair in mice by simvastatin mixed in the feed[14].

Although effective, the dose used in that study (about 100 times the recommended maximum clinical dose, as set out in the official label text) seemed impractical if statins were to have any use in bone formation in a clinical situation.

Most of the orally administered simvastatin in our previous study would have been sequestered in the liver, as only a few per cent of orally administered simvastatin reaches the general circulation in an unbound form and are accessible to extra-hepatic cells (Official label text, [15-18]). Consequently, in order to achieve a dose which would be clinically useful, we would need to by-pass this first pass clearance of the liver. We therefore conducted a number of experiments on fracture repair in which we administered the simvastatin as one daily subcutaneous injection in doses ranging from 1 to 100 mg/kg body weight. We were unable to find any significant effect of the statins with this setup (data not shown).

With one daily injection, the concentration of simvastatin would reach a peak relatively quickly, and then leave the organism. The elimination half-life of simvastatin is about 2 hours in humans, and probably not longer in mice[19,20].

Consequently, two questions arose. Firstly, is a continuous plasma concentration necessary for an effect on fracture repair? If so, subcutaneous injections would not work, whereas a continuous subcutaneous release of simvastatin would yield positive results similar to the ones achieved when mixing it in the feed. Secondly, since the effect of simvastatin on bone metabolism seems to be a local effect on bone cells; would local delivery to the fracture work?

In order to answer these questions, we performed three experiments. Firstly, we conducted an expanded experiment with subcutaneous injections. Secondly, subcutaneously implanted osmotic mini-pumps were used to deliver a continuous systemic dose of simvastatin. Thirdly, silicone tubes were led subcutaneously from implanted osmotic mini-pumps to the fracture area, delivering a local continuous dose.

## Methods

70 mature male Balb-C mice were used. The study had been approved by the regional ethics board and institutional guidelines for the care and treatment of laboratory animals were adhered to. The mice were kept 1 per cage with free access to mouse-chow and water.

Simvastatin powder (kindly supplied by MSD) was dissolved in PEG 400 (Sigma-Aldritch Chemie Gmbh, Steinheim, Germany) and passed through a sterile filter (Millex™, pore-size 0.22 μm, Millipore Corporation) before injection or filling the mini-pumps.

### Surgical procedure

The mice were anesthetized with isoflourane gas. Each mouse received a preoperative subcutaneous injection of 1.5 mg oxytretracycline and 0.003 mg of buprenorphine. Implants and surgical equipment were sterilized in an autoclave. Sterile gowns, gloves, surgical masks and theatre caps were used. The mouse leg on the operated side was shaved and the entire mouse was put into a sterile surgical glove. Subsequently, a hole was cut out of the glove and the leg pulled out through the hole using tweezers and the leg washed with chloro-hexidine alcohol.

**Figure 1:**
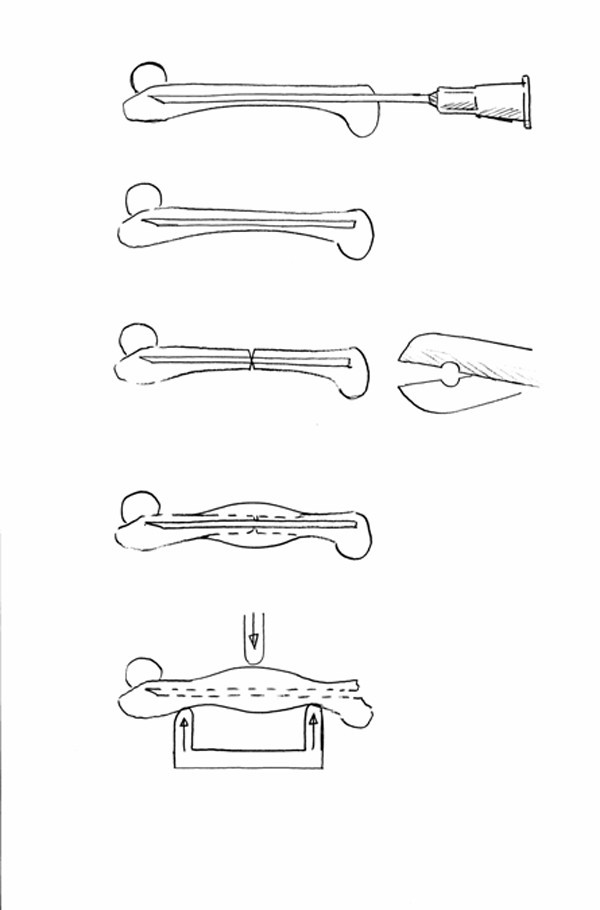
Overview of experimental set-up. After insertion of the intramedullary cannula,, the specially made pair of scissors with semi-lunar cutting edges was slid along the bone to approximately mid-diaphysis and the femur cut.

**Figure 2:**
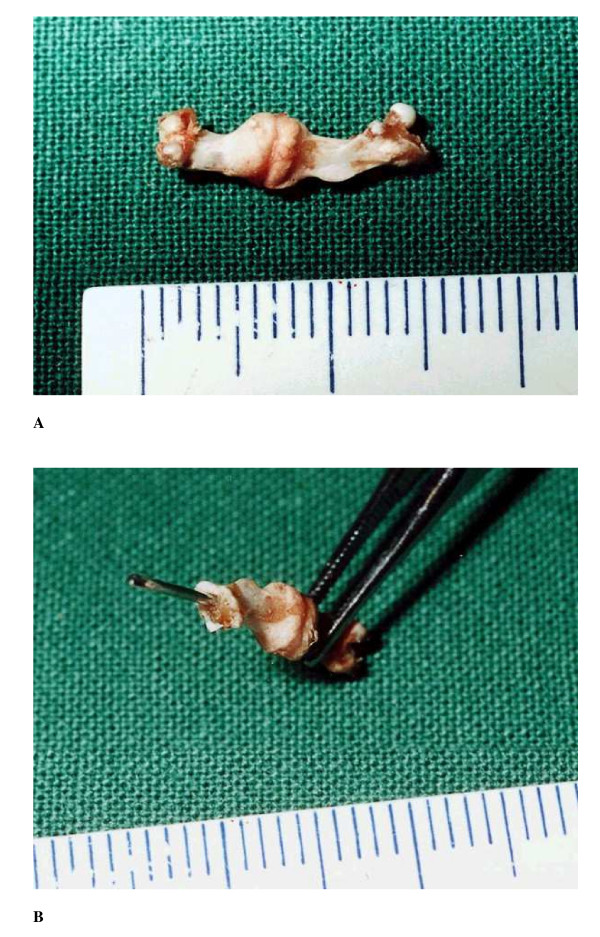
Photographs taken at evaluation to illustrate the three-point bending.

**Figure 3:**
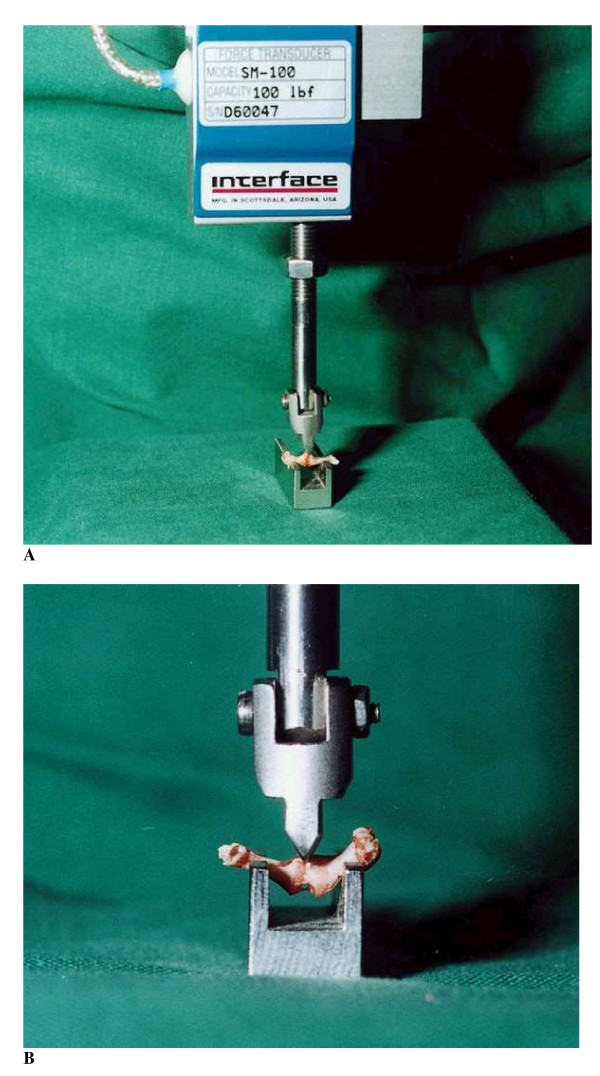
Photographs of fractured femurs taken at 14 days post-operative. Figure 3b shows positioning of the intramedullary cannula.

A lateral incision was made along the distal femur. The patella was dislocated medially with the blunt side of the scalpel, so that the femoral condyles were exposed. Using an intercondylar approach, a hole was drilled through the medullary canal of the femur using a cannula (diameter 0.4 mm). A wider cannula (0.6 mm) was then inserted into the canal made. The sharp end of the cannula was blunted in order not to penetrate through to the hip. The cannula was cut off, so that the remaining bit was inside the bone and no end extended beyond the bone. A specially made pair of scissors with semi-lunar cutting edges were then slid along the bone to mid-diaphysis and the femur cut to produce a fracture (Figures 1, 2 &3). The patella was then repositioned over the knee-joint and the muscles and skin sutured separately.

### Systemic treatment, subcutaneous injections

20 mice were used. The simvastatin powder was dissolved in PEG-400 so as to ascertain a concentration of 1 mg/ml. Starting on the day of surgery, subcutaneous injections of approximately 20 mg per kg body weight per day were administered in the scruff to 10 mice for 14 days. The remaining 10 mice received vehicle injections.

### Systemic treatment, osmotic mini-pumps

30 mice were used. On the day of surgery, the simvastatin powder was dissolved in PEG-400 so as to ascertain the proper concentrations. In the same session as when producing the fractures, Alzet osmotic mini-pumps (B & K Universal AB, Sollentuna, Sweden) for continuous release of 0.25 microL per hour were subcutaneously implanted in the scruff. Treatment was blinded to the operator and randomised before surgery by drawing lots. Treatment was divided into 3 groups of 10 mice, receiving the vehicle substance (PEG-400), the vehicle with 5 mg (c = 20.8 mg/ml) or with 10 mg (c = 41.6 mg/ml) of simvastatin per kg bodyweight every 24 hours for 14 days.

### Local treatment, osmotic mini-pumps

20 mice were used. The simvastatin powder was dissolved in PEG-400 so as to ascertain a concentration of 0.416 mg/ml. A silicone tube was subcutaneously led from the implanted osmotic mini-pump to the fracture area and fixed in place with a single 6.0 monofil suture in the adjacent muscle. Treatment was randomised before surgery by drawing lots. The mini-pumps had a release profile which meant that approximately 0.25 microL was released per hour and 0.1 mg of simvastatin per kg bodyweight was released to the area every 24 hours for 14 days. In this way, 10 mice received a local dose of simvastatin for the duration of the experiment and 10 mice received the vehicle substance.

### Evaluation

The mice were sacrificed at 14 days. Bilateral femurs were harvested, the marrow-nail extracted and the maximal sagittal and transverse diameters of the callus and the mid-diaphysis of the unoperated femurs were measured with a digital calliper. Biomechanical tests were performed by way of three-point bending (beam length 6 mm) in a computerised machine (100 R, DDL Inc., Eden Praire, Mn, USA) (Figures 3a &3b). The bending force was applied in the sagittal plane. Mechanical data evaluated were force at failure (N), energy (Nmm) until 10% droop from maximum force along the load- deformation curve and Young's modulus of elasticity (MPa). All evaluations were performed while blinded as to treatment.

For the subcutaneous injections, results were analysed with Student's t-test. For the systemic mini-pump experiment, statistical analysis was carried out with ANOVA, followed by Scheffé's post-hoc analysis for comparisons between groups. For the local treatment, results were analysed with Student's t-test.

## Results

The mice recovered well after surgery and did not display overt signs of discomfort. Weight-bearing on the operated leg began within the first post-operative week.

In the subcutaneous injections experiment, 2 mice were excluded because of faulty placement of the intramedullary pins. In the systemic continuous release experiment, 4 mice were excluded. One due to failed surgery, one due to death shortly after surgery and 2 due to technical errors while harvesting the femurs. In the local treatment experiment, one femur was excluded due to faulty intramedullary pin placement. Exclusions were done while blinded.

### Subcutaneous injections

No effects could be demonstrated for any of the parameters examined (Table 1).

**Table 1:** Three-point bending (beam length 6 mm) of healing mice femur fractures at 14 days treated with daily subcutaneous injections of simvastatin

Treatment	n	Days	Force at Failure (F)		Energy Uptake (Nmm)		Area (mm^2^)		Young's modulus (MPa)	
			m	sd	m	sd	m	sd	m	sd

Simvastatin	10	14	4.4	1.9	1.6	0.9	11.1	2.4	666	1440
Control	8	14	4.1	1.3	1.7	1.0	11.3	2.2	437	705
p			0.68		0.88		0.82		0.69	

M signifies mean, SD signifies standard deviation.

### Continuous systemic delivery

Simvastatin (5 mg/kg/day) resulted in a 160 per larger force at failure and an insignificant trend towards increased modulus and energy (Table 2). Biomechanical effects could not be found for the contra lateral, unbroken femurs.

**Table 2:** Three-point bending (beam length 6 mm) of healing mice femur fractures at 14 days treated with continuous subcutaneous administration of simvastatin

Treatment	n	Force at Failure (F)		Energy Uptake (Nmm)		Area (mm^2^)		Young's modulus (MPa)	
		m	sd	m	sd	m	sd	m	sd

Simvastatin (5 mg)	8	5.1	1.5	2.4	0.9	11.2	1.5	201.8	128.8
Simvastatin (10 mg)	8	4.1	1.4	1.7	0.7	10.9	1.7	231.3	221.2
Control	10	3.2	1.6	1.9	1.1	9.8	2.3	163.5	67.3
p*		0.04		0.6		0.3		0.9	

* Signifies the p value for Scheffe's post-hoc test when comparing simvastatin 5 mg with controls. ANOVA showed a significant difference between groups (p = 0.04) M signifies mean, SD signifies standard deviation.

### Local delivery

For the operated femurs, simvastatin treatment resulted in a 170% larger force at failure as well as a 200% greater energy uptake. Callus size was not significantly affected. Modulus of elasticity displayed a trend towards higher values as a result of the simvastatin, but this was not significant (Table 3). No significant biomechanical effects could be found for the contra lateral, unbroken femurs.

**Table 3:** Three-point bending (beam length 6 mm) of healing mice femur fractures at 14 days treated with continuously, local administration of simvastatin

Treatment	n	Force at Failure (F)		Energy Uptake (Nmm)		Area (mm^2^)		Young's modulus (MPa)	
		m	sd	m	sd	m	sd	m	sd

Simvastatin	9	7.2	2.0	3.3	1.0	11.2	1.7	635.7	647.1
Control	10	4.5	1.3	1.7	0.9	10.4	1.2	278.4	130.8
p		0.003		0.002		0.24		0.1	

M signifies mean, SD signifies standard deviation.

## Discussion

In contrast to our previous results, we could not demonstrate an effect on cross-sectional area. This, combined with a general increase in biomechanical parameters, seems to point to the possibility of improved material properties, indicating an increased callus maturity with more bone. Such an increase would be in line with several in vitro studies, which have reported an increase in osteoblast differentiation and calcification by statins[4-6,21-25]. We could not demonstrate improved maturity by histology in our previous study with orally administered simvastatin. The difference between that study and the present one could be due to different local doses (as has been suggested as a possibility by Maritz et al[12]) or a failure to detect actual effects.

Enhanced repair by local simvastatin has been demonstrated in a critical size defect model in rabbits[9,11], and there are plentiful *in vitro *data which support an anabolic effect on bone by statins [5,23,26-30]. Furthermore, it seems that, at least for this mice model, a sustained continuous release is necessary for an effect. From our experiments, it seems clear that the *in vivo *effects of simvastatin on bone is a local phenomenon not related to the established cholesterol-lowering effect, and that a local delivery system can be as effective as systemic treatment in promoting fracture healing.

From personal communication with Mundy's group, we had learned that above a certain dose, the statins did not have any effect on bone in their experiments. Further, indirect evidence for a possible biphasic effect on bone comes from research into possible angiogenesis effects of statins[31], and there seems to be quite a flat dose-response curve[32]. Therefore, to get the desired effect, one should perhaps maintain a certain local concentration of the drug over a longer period of time. This is what we most probably achieved in our previous study by mixing the drug in the feed, since mice eat more or less continuously throughout the day. In contrast, and similar to subcutaneous injections, gavage should lead to a high but relatively brief concentration peak of the drug. Such a regimen has been reported to have little effect in mice [13].

## Conclusion

In conclusion, we found an effect of simvastatin treatment directly applied to the fracture area. Our current results are in parity with what we saw in the first study with orally administered simvastatin[14]. In that study, the required doses were excessive. By using local treatment we were able to reduce the total dose by three orders of magnitude. The resultant negligible systemic levels bring us one step closer to the feasibility of using statins to improve fracture healing in a clinical setting. A natural next step would be to deliver the simvastatin coated to the implants, as we have previously successfully done with bisphosphonates[33].

## Competing interests

Björn Skoglund received financial recompense for some of the work performed on this study from Synthes, Basel.

## Authors' contributions

BS and PA conceived of the study, participated in the design of the study and performed the statistical analysis. BS carried out the experiments and drafted the manuscript with the help of PA. Both authors read and approved the final manuscript.

## Pre-publication history

The pre-publication history for this paper can be accessed here:


